# IgD multiple myeloma a descriptive report of 17 cases: survival and response to therapy

**DOI:** 10.1186/1756-9966-31-17

**Published:** 2012-03-01

**Authors:** Francesco Pisani, Maria Teresa Petrucci, Diana Giannarelli, Velia Bongarzoni, Marco Montanaro, Valerio De Stefano, Giacinto La Verde, Fabiana Gentilini, Anna Levi, Tommaso Za, Alessandro Moscetti, Luciana Annino, Maria Concetta Petti

**Affiliations:** 1 Department of Hematology, Regina Elena National Cancer Institute, Rome, Italy; 2 Department of Cellular Biotechnologies and Hematology, "Sapienza" University of Rome, Rome, Italy; 3 Department of Hematology, S.Giovanni-Addolorata Hospital, Rome, Italy; 4 Department of Hematology, Belcolle Hospital, Viterbo, Italy; 5 Department of Hematology, Catholic University, Rome, Italy; 6 Department of Hematology, Sant'Andrea Hospital "Sapienza" of Rome, Rome, Italy

## Abstract

**Background:**

Immunoglobulin D multiple myeloma (MM) is rare and has a poorer prognosis than other MM isotypes.

**Design and methods:**

Seventeen patients (pts) diagnosed from 1993 to 2009 with IgD MM were selected from six institutions of Multiple Myeloma Latium-Region GIMEMA Working Group.

**Results:**

Median age was 55 years, 14 patients had bone lesions, eight had renal impairment with estimated glomerular filtration rate (eGFR) < 50 ml/min, one serum calcium ≥ 12 mg/dl, 11 had lambda light chains, five stage III of ISS, six with chromosomal abnormalities. Six pts received conventional chemotherapy (CT): five melphalan + steroids based regimens. Eleven underwent high-doses of chemotherapy with autologous stem cell transplantation (HDT/ASCT), five single and six tandem ASCT: six received bortezomib and/or thalidomide as induction therapy and five VAD. Thalidomide maintenance was used in two pts: one in HDT/ASCT and one in CT group; bortezomib was used in one patient after HDT/ASCT. At a median follow up of 38 (range 19-60) and 50 months (range 17-148) for pts treated with CT and HDT/ASCT, respectively, the overall response rate (ORR) was 83% and 90%. In the group of patients treated with CT, median overall survival (OS) was 34 months (95% CI 15- 54 months), median progression free survival (PFS) was 18 months (95% CI 3-33 months) and median duration of response (DOR) was 7 months (95% CI 5-9 months). Median OS, PFS and DOR were not reached at the time of this analysis in the HDT/ASCT group of patients. Death was observed in 27.3% of pts treated with HDT/ASCT and in 66.7% undergone CT.

**Conclusions:**

Despite the retrospective analysis and the small number of pts our study showed that the use of HDT/ASCT seems to improve also the prognosis of IgD MM patients. Treatment options including new drugs, before and after stem cell transplantation, may further improve the outcomes of these patients.

## Background

Immunoglobulin (Ig)D multiple myeloma (IgD MM) is a rare subtype of myeloma, accounts for less than 2% of all myelomas [1] and is accompanied with aggressive course, resistance to chemotherapy and poor outcome. It is often associated with relatively high frequencies of renal failure, extra osseous disease, hypercalcemia, amyloidosis and Bence-Jones proteinuria [2-5]. The survival of patients with IgD MM has been reported to be shorter than that of patients with other types of M-protein [2,4,6]. However, there are reports of long survival in patients with IgD MM treated with alkylating drugs [7-9], interferon-alfa monotherapy [10] or autologous stem cell transplantation (ASCT) [11-15]. Over the past 10 years, there has been substantial progress in the treatment of MM, prospective randomized trials have shown the superiority of high-doses of chemotherapy with autologous stem cell transplantation (HDT/ASCT) over standard therapy (CT) and new drugs, such as immunomodulatory agents and proteasome inhibitors, have shown effectiveness against disease. These developments may have led to changes in the outcomes of IgD MM.

In this report we present the results of a retrospective analysis of 17 cases with IgD MM treated with CT or HDT/ASCT in six institutions of Multiple myeloma Latium-Region GIMEMA Working Group between 1993 and 2009.

## Design and methods

A retrospective analysis was carried out of 17 patients with IgD MM diagnosed from 1993 to 2009 in six institutions from Multiple Myeloma Latium-Region GIMEMA Working Group. Patients who had overt MM based on International Myeloma Working Group (IMWG) diagnostic criteria were selected.

### Definition of response

Disease response was assessed using the IMWG criteria [16,17]. Briefly, a partial response (PR) was defined as a 50% or higher decrease in the serum monoclonal protein (M-protein) levels from baseline and a reduction 90% or greater in 24 hour urine M-protein excretion or less than 200 mg/24 hours; a very good partial respone (VGPR) required a 90% or greater reduction in serum M-protein and urinary M-protein less than 100 mg/24 hours or M-protein detectable by immunofixation but not by electrophoresis. A complete response (CR) was defined as negative serum and urine immunofixation and less than 5% plasma cells on bone marrow examination. Disease that did not satisfy the criteria for PR, VGPR, CR or progressive disease was classified as stable disease (SD). Disease progression required any of the following: 25% or greater increase from lowest response value in serum M-protein (absolute ≥ 0.5 gr/dl) or urine M-protein (absolute ≥ 200 mg/24 hours).

Progression free survival (PFS) was calculated from start of first treatment to disease progression or death from any cause, or the date the patient was last known to be in remission. Overall survival (OS) was calculated from start of first treatment to the date of death or the date the patient was last known to be alive. Duration of response (DOR) was calculated from the time of first response achievement, that is at least PR, to the time of disease progression, with deaths owing to causes other than progression not counted, but censored.

For the analysis of treatment responses, PFS and OS, the patients were divided into two groups: one group was treated with HDT/ASCT, the other group received treatment with conventional-dose chemotherapy.

### Statistical analysis

Data cut-off was April 2011; patient characteristics were summarized using descriptive statistics such as median and range for quantitative variables and frequencies for qualitative.

Progression free survival, overall survival and duration of response were estimated according to the Kaplan-Meier method. We used the Cox proportional hazards regression model to estimate hazard ratios and 95% CIs. Differences between survival curves were tested for statistical significance with the two-sided log-rank test.

### Patients

A total of 17 patients with IgD MM was identified, patients characteristics are listed in Table 1. The median age of the patients was 55-years (range 37-78); 8/17 patients had ECOG performance scores > 2 and 14 had ≥ 1 lytic bone lesions. Eight patients (47%) had renal impairment with estimated glomerular filtration rate (eGFR) < 50 ml/min, one patient had hypercalcemia (serum calcium concentration ≥ 12 mg/dl), 11 patients had lambda light chains (64%) and Bence-Jones proteinuria in 70%. Five patients were of stage III according to ISS; cytogenetic analysis by fluorescence in situ hybridization (FISH) was possible in six of eleven patients and the abnormalities are shown in Table 2. Only one patient was positive for amyloidosis at baseline.

**Table 1:** Patient characteristics at diagnosis

	Number of patients = 17
**Male/Female**	**11/6**

Median Age at diagnosis yr (range)	55 (37-78) years
	≤ 65 y = 13 (76.5%), ≥ 65 y = 4 (23.5%)
ISS stage at diagnosis	
I	7
II	2
III	5
Unknown	3
Performance status	
ECOG < 2	9
ECOG > 2	8
Light chain type	
k	6
λ	11

Bone marrow infiltration	30% (10-70%)
Extra osseous disease	0
Bone lesions	14/17 (82%)
Median serum monoclonal protein g/dl	1.05 (0.09-5)
Median Urine monoclonal protein g/24 h	0.79 (0-28)
Urine immunofixation positive	12/17 (70%)
Serum β2 microglobulin > 5.5 mg/L	5/17 (29%)
Serum albumin < 3.5 g/dl	5/17 (29%)
eGFR < 50 ml/min	8/17 (47%)
Serum Calcium > 12 mg/dl	1/17
Amyloidosis	1/17

Hemoglobin g/dl, median (range)	11.9 (6.5-15)
< 10	5/17 (29%)
WBC count 10^9^/L, median (range)	6.57 (3.19-16.8)
> 7 × 10^9^/L	7/17 (41%)
Platelet count 10^9^/L, median (range)	214 (74-518)
< 100 × 10^9^/L	1/17

**Table 2:** Interphase FISH cytogenetic profile results

	Number of patients = 17
Not available	11

Available	6

del(13q)	1/6

del(6q)	1/6

t(11;14)	2/6

-Y	1/6

+11	1/6

## Results

Six patients were treated with CT, five with Melphalan plus steroids based regimens and one with VAD (Vincristine, Adriamycin and Dexametasone) plus CED (Cyclophosphamide, Etoposide, Dexamethasone); one patient showed a CR, two VGPR, two PR and one SD. Thalidomide was used as maintenance in the patient who obtained CR after CT. The overall response rate (ORR) was 83%; after a median follow up of 38 months (range 19-60) for patient treated with conventional chemotherapy, the median OS was 34 months (95% CI 15- 54 months) and the median PFS was 18 months (95% CI 3-33 months). Median DOR was 7 months (95% CI 5-9 months).

**Figure 1:**
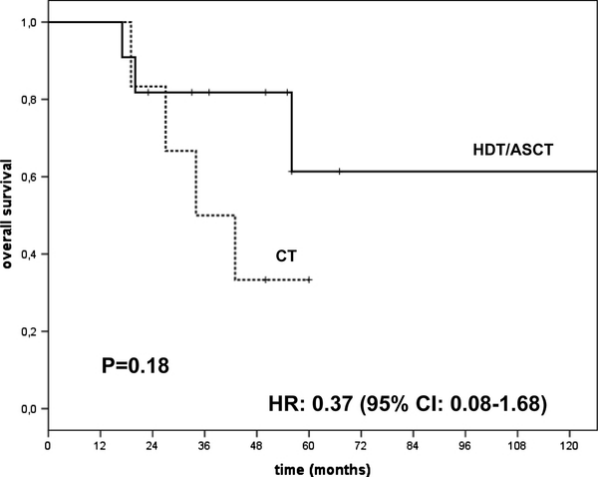
**Overall Survival of HDT/ASCT and CT groups**.

**Figure 2:**
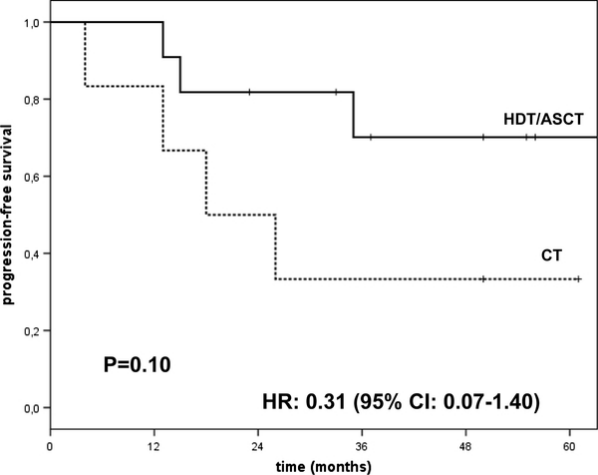
**Progression free survival of HDT/ASCT and CT groups**.

Eleven patients underwent HDT/ASCT, as part of their front line therapy, five patients received single and six tandem ASCT. Six out of 11 patients received induction chemotherapy including thalidomide and/or bortezomib and five VAD. After ASCT single or tandem, five patients obtained CR, three VGPR, two PR and one SD. One patient in PR after HDT/ASCT received maintenance with bortezomib and another patient also in PR received Thalidomide as maintenance treatment both patients maintained PR. The ORR in patients treated with HDT/ASCT was 90% after a median follow up of 50 months (range 17-148); median OS, PFS [Figures 1, 2] and DOR are not reached. The log-rank test for DOR was *P *= 0.23.

A progression or relapse was observed in 4/11 (36.4%) patients treated with HDT/ASCT and in 4/6 (66.7%) of those undergone CT. The log-rank test for PFS was *P *= 0.10, the hazard ratio was 0.31 (95% CI 0.07-1.40). One patient who received single ASCT was treated with allogeneic transplantation at relapse. Peripheral neurophaty of grade 1-2 was observed in all patients treated with thalidomide and or bortezomib either in induction or in maintenance therapy. All patients with bone disease received bisphosphonates; patients treated with thalidomide, received aspirin or low molecular-weight heparin as thromboprophylaxis and nobody developed venous thromboembolism.

Seven of 17 patients had died by the time of analysis: four in the group treated with CT and three in the group of HDT/ASCT, 85% of death for disease progression; there were no peritransplant deaths. Comparing OS with log-rank test we obtained *P *= 0.18, the hazard ratio was 0.37 (95% CI 0.08-1.68). FISH analysis was available only for 6/17 of cases, in these six patients cytogenetic profile had not statistical significance for OS, PFS or DOR

## Discussion

The clinical features of our patients reported in this study underline the worse characteristics of IgD MM. As in other series described in the literature [18], we also found an advanced stage and a younger age at presentation, with more aggressive clinical course. In addition, the poor survival of the patients may be associated with problems related to delayed diagnosis [13,19].

Patients with renal failure of unknown cause, bone pain, small serum M-protein bands, or unidentified Ig isotype should be suspected for IgD MM. However, the underlying tumor biology responsible for the differences between IgD MM and other MM isotypes remains to be defined.

IgD MM should be considered a rare subgroup of MM with aggressive features rather than a single parameter of poor prognosis.

Jancelewicz et al. [2] reported that λ light-chains are found in 90% and almost the totality of patients had Bence-Jones proteinuria. A mean survival of 13.7 months from diagnosis, that was worse than the common myelomas, was observed in this study. Bladé et al [4] reviews outcomes in 53 patients from 1965 to 1992 and observed λ light-chain disease in 60%, Bence Jones proteinuria in 96%, renal failure in 33% and hypercalcemia in 22%. Shimamoto et al [6] reviewed 165 cases with IgD MM and on multivariate analysis showed that λ light-chains and white blood cell count > 7 × 10^9 ^where adverse prognostic markers. In our series we registered in 11 out of 17 patients (64%) the presence of λ light-chains and Bence-Jones proteinuria in 70%, renal impairment with eGFR < 50 ml/min in 8 cases (47%), extra osseous disease was not seen in our patients at diagnosis. Many studies have shown a poorer prognosis of IgD myeloma than other MM isotypes. Bladé et al. [4] observed an overall response to therapy of 58% with a median overall survival of 21 months and 5-years survival was 21%. However, these results were obtained before the use high-dose therapy. Wechalekar et al in 11 cases IgD myeloma treated with autologous stem cell transplantation reported 18% CR and 82% PR, compared with a group of 14 patients who received conventional chemotherapy alone in which was observed 0% CR and 43% PR. Maisnar et al [13] reviewed 26 cases with IgD MM; ten were treated with first-line high-dose chemotherapy using melphalan 200 mg/m^2 ^followed by ASCT and 70% achieved a CR and 100% had at least a PR. The median PFS was18 months for patients who received ASCT and 20 months for those who received conventional chemotherapy. However, the median OS for ASCT group had not been reached, in contrast the median OS for chemotherapy group was only 16 months, which was statistically significant (*P *= 0.005). More recently Kim et al [17] retrospectively reviewed 75 patients with IgD myeloma from the Korean Myeloma Registry data base; among 34 patients (45%) treated with ASCT who were in CR or PR, after induction therapy, had a median OS of 30 months (95% CI 17.7-42.3 months) significantly longer than that of patients treated with conventional chemotherapy (16.4 months, *P *= 0.012).

## Conclusions

The small group of patients suffering from IgD multiple myeloma is rare and considered to have a poor prognosis compared to other MM isotypes. Our report, based on analysis of a cohort of 17 patients treated over two decades in six institutions, shows that the use of HDT/ASCT increased OS and PFS by 63% and 69%, respectively, in comparison with those of patients treated with conventional chemotherapy. Thus, the advantage of HDT/ASCT over conventional chemotherapy seems confirmed, although the small number of patients limited the statistical power of the analysis.

New drugs, such bortezomib, thalidomide, lenalidomide used as induction and consolidation in the stem cell transplantation program, may well improve the outcomes of IgDMM.

The clinical features and prognosis of patients with IgDMM differ from those that characterize patients with other immunoglobulin MM subtypes. The underlying tumor biology responsible for these differences remains to be determined. New treatment strategies that aim to induce high-quality responses before ASCT and maintain the response after ASCT may be needed to improve the outcomes of such patients.

## Abbreviations

MM: Multiple myeloma; pts: Patients; eGFR: estimated Glomerular Filtration Rate; CT: Conventional Chemotherapy; HDT/ASCT: High-dose chemotherapy with autologous stem cell transplantation; VAD: Vincristine Adriamycin Dexametasone; ORR: Overall Response Rate; OS: Overall Survival; PFS: Progression Free Survival; DOR: duration of response; Ig: Immunoglobulin; IgDMM: IgD Multiple Myeloma; IMWG: International Myeloma Working Group; M-protein: monoclonal protein; PR: Partial Response; VGPR: Very Good Partial Response; CR: Complete Response; SD: Stable Disease; CED: Cyclophosphamide Etoposide Dexametasone; ISS: International Staging System; FISH: Fluorescence in situ Hybridization.

## Competing interests

The authors declare that they have no competing interests.

## Authors' contributions

Conception and design: FP wrote the paper. MPT and VDS have been involved in drafting the manuscript and revising it critically. DG has made statistical analysis. Provision of study materials or patients: FP,MPT,VB,VDS,GLV,FG,AL,TZ, AM,LA,MCP. All authors have read and approved the final manuscript. 
